# How Information-Seeking Behavior, Essential Technologies, and Resilience Enhance the Academic Performance of Students

**DOI:** 10.3389/fpsyg.2021.651550

**Published:** 2021-08-26

**Authors:** Muhammad Miraj, Lu Chuntian, Ridzwana Mohd Said, Robert Osei-Bonsu, Ramiz ur Rehman

**Affiliations:** ^1^School of Humanities and Social Science, Institute for Empirical Social Science Research (IESSR), Xi'an Jiaotong University, Xi'an, China; ^2^School of Business and Economics, Universiti Putra Malaysia, Serdang, Malaysia; ^3^Department of Biblical and Theological Studies, Adventist University of Africa (AUA), Nairobi, Kenya; ^4^Lahore Business School, The University of Lahore, Lahore, Pakistan

**Keywords:** academic performance, information-seeking behaviors, IT skills, reading/writing abilities, resilience

## Abstract

This study explores how the scholarly accomplishments of students might increment due to specific fundamental causes. The academic performance of the student was prioritized as a dependent variable, and the independent indicators chosen were “information seeking, IT ability, reading/writing capacity, and resilience.” At the same time, age, gender, marital status, and family income were included in the control variables. The research subject samples were limited to (*N* = 288) postgraduate students from three mega universities in Islamabad, Pakistan. Forward regression analysis was performed in this research to decide the impact of the indicators. The results indicate that information seeking affects academic performance positively and significantly. Essentially, the study revealed that information technology (IT) skills make a fundamentally positive and significant impact on academic performance. Reading and writing influenced academic performance considerably. In addition, resilience affected academic performance emphatically and essentially. Further, this research also noted the relationships between information-seeking behaviors, IT ability, reading/writing capacities, and resilience capabilities and the academic performance of students. These variables have a positive impact on the academic performance of students.

## Introduction

Academic performance is an avenue where students can showcase their talents and be recognized for their achievements. Students who excel academically in school are often aware that they have the intellectual ability to achieve their academic goals. Furthermore, students who are excellent academically have the ability to communicate and articulate their views in class. Academically superior students also have the perception that they can measure up to others intellectually. When intelligent students enroll in a new course, they usually expect to be among the top 25% of students in the class. When brilliant students face critical examinations or other challenging learning experiences, they typically believe that they will sail through, counting their intellectual abilities. Moreover, academically superior students usually seek out those activities that are intellectually challenging because they know they can do them better than most people. Numerous studies have been conducted to assess academic performance in diverse situations using various factors. For instance, research in Taiwan based on survey data from 295 vocational high school students found that students with poor academic self-efficacy gain more from internet information seeking in terms of academic performance (Zhu et al., 2011). In the context of information technology (IT) skills, a cross-sectional study of final-year undergraduate students at the University of Professional Studies in Accra, Ghana, found that information and communication technology (ICT) might be used to improve academic performance at the university level (Nketiah-Amponsah et al., 2017). Similarly in the context of reading/writing skills and their impact on student performance, a study based on data from first- and second-year undergraduate psychology students at a university in UK revealed that both self-efficacy of reading and self-efficacy of writing were connected to actual writing performance. Overall, their findings confirm the significance of the notion of self-efficacy in terms of student performance (Prat-Sala and Redford, 2012), while another study indicated that academic self-efficacy and resilience substantially impact academic performance and achievement (Unachukwu et al., 2020). The research mentioned above discovered the links between “information-seeking, IT abilities, reading/writing skills, resilience,” and the academic performance of students. The data used in these studies came from diverse areas, circumstances, and academic levels of students. Given the significance of these four elements and their advantages to the academic performance of students, we decided to undertake research focusing on these four aspects on a single platform. Hence, this study seeks to reveal the holistic academic performance of postgraduate students in Pakistani academic culture based on four factors: information seeking, IT skills, reading and writing abilities, and academic resilience capabilities.

This study is based on two theoretical concepts such as self-efficacy and life skills; we utilized a self-efficacy concept, which alludes to the confidence of people in their ability to perform activities (Bandura, 1977, 1986, 1997). Information-seeking behavior, IT skills, and reading/writing abilities have been chosen as the indicators to be explored in light of the self-efficacy theory. It is anticipated that these variables will influence the academic performance of students. Recent research, for example, found that information seeking, and therefore academic self-efficacy, mediates the positive relationship between exploration and academic performance (Gkorezis et al., 2017). A recent study found a connection between ICT adoption and academic performance in a conservative setting (Basri et al., 2018). As to the relationship between reading/writing and academic performance, the findings of a study indicate an association between reading abilities and academic performance (Lukhele, 2013).

Similarly, the academic resilience of students has been included in the study as a fourth indicator, with the expectation that it will affect the academic performance of students. The ability of an individual to cope with stress is a life skills concept announced by WHO, also known as resilience, which has much to do with the ability of an individual to succeed in academics (World Health Organization, 1999). For example, a study investigated the effects of self-esteem and resilience factors on the academic performance of international students vs. domestic Australian students. Their findings indicated that self-esteem and endurance are significant predictors of academic performance in both categories (Kwek et al., 2013).

Previous scholars evaluated academic performance using various criteria and in a variety of circumstances and standards. For example, student academic performance is an excellent concern for educational institutions in all levels of academic years (Alsalman et al., 2019). Similarly, a study demonstrates a significant difference in the academic performance of medical students as influenced by social media usage (Alnjadat et al., 2019). Another analysis indicates that cognitive skills significantly predicted the performance in chemistry, while psychomotor skills and everyday applications had no significant impact on the performance of students (Ramnarain and Ramaila, 2018). Structural equation modeling reveals that compassion partially mediates the relationship between mindfulness and engagement and, consequently, increases academic performance (Miralles-Armenteros et al., 2021).

On the contrary, a study found a negative factor in academic performance. For example, they indicated a negative and significant relationship between the widespread use of social networks and the academic performance of students (Azizi et al., 2019). Similarly, a Hong Kong study found that using social media for educational purposes was not a significant predictor of academic performance as measured by cumulative grade point average, whereas utilizing social media for non-academic purposes (video gaming in particular) and social media multitasking significantly negatively impacted the predicted academic performance (Lau, 2017). At the same time, another study demonstrated the positive and negative role of self-efficacy and academic performance. It indicated that self-efficacy might play supportive and protective roles by increasing the positive effect of mastery and performance-approach goals and reducing the negative impact of avoidance goals on academic performance, respectively (Alhadabi and Karpinski, 2020). Further research has shown that academic performances have a weak significant and negative association with multitasking preferences and media and technology usage, but not self-regulation control. Their hierarchical multiple linear regression analyses revealed that demographic variables, including gender, age, and year of study, were significant predictors of the academic performance of students. Additionally, after controlling these particular demographics, only media and technology usage significantly and negatively contributed to predicting the academic performance of students, whereas multitasking and control or self-regulation did not (Uzun and Kilis, 2019). A study found a significant relationship between the former educational background of students, studying hours, and the behavior of students on taking an alcoholic drug and chatting on the academic performance of students (Yigermal, 2017). The frequency of internet connection has been positively correlated with academic performance, whereas internet traffic volume features are negatively associated with academic performance (Xu et al., 2019). East Asia creates a higher level of disciplined atmosphere than other cultures, and student academic performance positively correlates significantly with the disciplinary climate (Ning et al., 2015; Guo et al., 2018).

The preceding studies assessed academic performance in a variety of contexts and used a variety of variables, while this study is significant in that it has applied four major variables on a single platform and revealed their favorable effects. We used the correlation and stepwise regression analysis to investigate the influence of the variables. Furthermore, age, gender, marital status, and family income were used as the control variables. Consequently, this study discovered four essential factors, namely, information-seeking abilities, IT-operating capacity, reading/writing experience, and resilience capability, which significantly impact the overall academic performance of students. Self-efficacy gave rise to the idea of believing in the abilities of an individual in terms of information seeking, IT use, and reading/writing skills. Similarly, life skills teach people how to cope with stressful situations, which is known as resilience. Thus, this research applied the variables that fall under these two frameworks and discovered their positive effects on academic performance in understanding Pakistani postgraduate students. This study presented policymakers with a clear pathway for focusing on these elements in higher education.

## Theoretical Background

The link between psychological resilience and self-efficacy in adolescents has been identified as one of the most significant subjects of developmental age-positive adjustment (Bandura, 1986; Sagone and De Caroli, 2016; Sagone and Indiana, 2017). This link recommended evaluating the function of perceived self-efficacy in life skills connected to resilient resources, defined as the capacity to overcome and thrive in the face of adversity (Ryff and Singer, 2003) and to surface in the face of adversity (Hawley, 2000). Different studies investigated the effects of information-seeking behavior, IT skills, reading/writing ability, and resilience on the academic performance of students individually. For example, the positive relationship between exploration and academic performance is mediated by information-seeking, which in turn is mediated by academic self-efficacy (Gkorezis et al., 2017). Similarly, in the context of digital literacy, the study found that technology indirectly influences academic performance through self-directed learning (Rashid and Asghar, 2016). There were significant positive connections between all measures of writing beliefs, particularly reading and writing self-efficacy. Writing beliefs were connected with deep learning and, to a lesser extent, strategic learning (Maguire et al., 2013). Planning useful programs for increasing academic resilience and academic self-efficacy, as well as providing an enabling environment, can improve student adjustment and academic performance (Sadoughi, 2018). Previous research has focused on academic performance with different components and circumstances. However, this study looked at information-seeking behavior, IT skills, reading and writing, and resilience effects on the academic performance of students in a single platform. This research utilized two theoretical concepts, namely, social cognitive and concept of life skills theories. For example, the social cognitive theory has been used to bolster the information-seeking behavior of students and their abilities through essential IT skills and reading/writing capacity. In contrast, the life skills framework has been utilized to bolster the academic resilience capacity of students.

### Social Cognitive Theory

Self-efficacy is described as the confidence and ability of an individual to organize and ensure that an action is carried out to produce results (Bandura, 1997). According to an examination of the motivating influences in information-seeking behavior, the more important the information-seeking activity, the more likely the actor is to begin looking for more information (Savolainen, 2012). He further indicated that self-efficacy, which has both cognitive and affective aspects, could be a powerful motivator in information-seeking behavior. Wilson defined information-seeking activity as the intentional search for information to achieve a specific purpose (Wilson, 2000). The searching process includes seeking and using sources and creating thoughts about a subject and the feelings that usually follow such thought evolution (Kuhlthau, 1988). Utilizing the concept of self-efficacy, we hypothesized that the academic performance of students who believe in their ability to seek the required scholarly information in an educational setting would be exceptional.

The concept of self-efficacy was set up within the setting of social cognition theory (Bandura, 1986). Self-efficacy refers to the beliefs of an individual in their capacity to perform activities required to create specific enhancements in results (Bandura, 1977, 1986, 1997). Self-efficacy of the computer assumes that one can perform a particular task using it (Bandura, 1997). The literature discusses that computers use dynamic tools to improve cognitive skills and helps the learner develop helpful learning strategies (Caldwell, 1980). Self-efficacy of the computer means a judgment on the efficiency of using a computer (Compeau and Higgins, 1995). This study picked the fundamental IT abilities of students as an independent variable. Regarding another autonomous factor of the study, i.e., IT skills, we proposed that if students believe in utilizing their IT abilities in an educational setting that might increase the academic performance of students.

The idea of reading and writing skills as a sense of self-efficacy is problematic and limited. However, various studies have found a link between self-efficacy in reading and writing ability. For example, the study discovered a connection between writing self-efficacy and writing performance among undergraduate teacher candidates (Pajares and Johnson, 1994). Similarly, the self-efficacy of students was closely linked to their reading achievement than to writing achievement; however, reading beliefs were better predictors of reading and writing achievement than writing beliefs (Shell et al., 1989). Self-efficacy about higher effectiveness in writing increases the likelihood of prison education, while actual reading and spelling skills do not predict participation (Jonesa et al., 2013). The findings of this study demonstrate the importance and domain-specificity of interest and self-efficacy in reading and writing for students who have persistent specific learning disabilities in literacy (Abbott et al., 2017). It has been suggested that a screening procedure for reading and spelling difficulties includes an assessment of self-efficacy in reading and writing (Jones et al., 2012). Beliefs for reading were more highly related to comprehension skills relative to component skills, whereas beliefs for writing were more highly related to component skills relative to communication skills (Shell et al., 1995). Self-efficacy of people refers to their specific judgments and beliefs about their abilities, such as reading a book or writing a poem (Walker, 2003). Hence in the light of the above studies, we anticipated that students who believe in their reading and writing abilities in a scholastic sense would do better academically than students who do not believe in their reading and writing abilities.

### Concept of Life Skills

The WHO defined five essential areas of life skills that it believed were applicable across cultures: (a) decision-making and problem-solving, (b) creative and critical thought, (c) communication and interpersonal skills, (d) self-awareness and empathy, and (e) dealing with emotions and tension (World Health Organization, 1999). Life skills are described as “those abilities that allow individuals to excel under the varied environments they live in, such as school, home, and neighborhood. Life skills may be behavioral (effective communication with friends and adults) or cognitive (effective decision making); emotional (assertiveness) or intrapersonal (goal setting)” (Danish et al., 2004). Life skills education is explained as “the facilitation of the learning of psychosocial skills needed to cope with the demands and challenges of daily life” (World Health Organization, 1999). We obeyed the life skills concept mentioned by the WHO, where they indicated one fundamental factor: coping with feelings and stress, known as resilience abilities. In this way, we hypothesized that students who can cope with scholastic stress could perform well-academically, whereas students who are not resilient cannot perform well-academically.

## This Study

**Figure 1:**
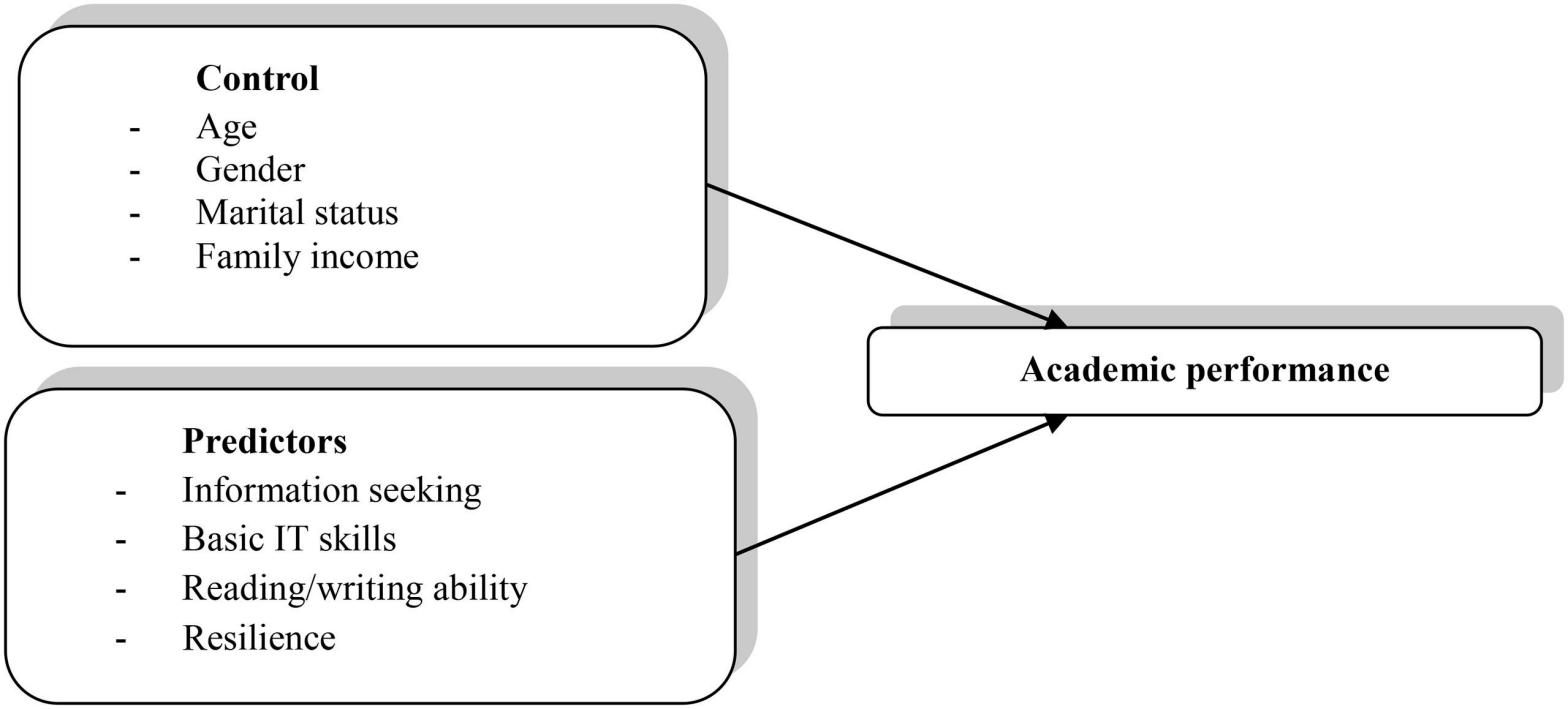
Conceptual model of predictors, control, and dependent variables.

Based on a single research query, we pursued this research about how information-seeking activity, hard IT expertise, reading and writing ability, and resilience will lead to the academic performance of students. The dependent, autonomous, and control variable directions are depicted in the conceptual model (see Figure 1). The academic performance of students has been selected as the dependent variable. At the same time, information-seeking behavior, basic IT skills, reading/writing ability, resilience, age, gender, marital status, and family income are chosen as the independent variables.

### Predictors

#### Information Seeking

Students with solid information-seeking capacity consider themselves to be exceptionally great at looking for and retrieving information. If they require information that they do not have, they know where to hunt for it and seek a solution to complicated issues. Within the scope of the information-seeking behaviors, this examination anticipated that those students would perform extraordinary well-academically because they can seek precise information to handle complex educational issues. Past literature indicated that information-seeking activity is characterized as the amount of all activities typically performed by higher education students to gather, use, and process any type of information required for their studies (Halder et al., 2020). Similarly, another study indicated that self-reported inattention symptoms, all components of meta attention, and domain experience predicted specific variation in online information-seeking capacity (Burek and Martinussen, 2021). A study looked at the relationship between health literacy, eHealth literacy, and actual online health information-seeking behavior. Their results show that even those who are eHealth literate cannot always use successful online browsing techniques. Further, they reported that the association between health and eHealth literacy scores was not statistically significant (Quinn et al., 2017). The Google search engine is a popular way to seek information (Chanda, 2021). A research discovered a complete mediation impact of information assessment on the relationship between information seeking desire and information choice decision-making for academic and life event-related activities (Chiu and Chan, 2017). Individuals with a strong level of conscientiousness perform faster in most information-seeking tasks (Al-Samarraie et al., 2017). Gender differences play an essential role in the knowledge behavior of university-level academic scholars. Gender and “time spent online for information seeking” have a clear correlation (Khan and Nisa, 2017). The tremendous development and widespread use of new ICT have affected information consumers worldwide. A digital environment of this magnitude has dramatically altered the information-seeking habits of information consumers in any culture (Das and Jadab, 2017). The motivation of students is the most critical pillar for seeking information while writing papers and performing studies (Amiramini Khalafloo and Bayat, 2018). Search engines continue to be the preferred information source for undergraduate students, with more than half of them rating search engines as authoritative, reliable, open, and appropriate (Okocha and Owolabi, 2020). A popular strategy for finding information is to use the Google search engine. The year and course of study influence the information need and seeking behavior of students (Eke et al., 2019).

#### IT Skills

The individuals are considered computer literates and proficient if they know how to use a computer to navigate spreadsheets, databases, PowerPoint presentations, statistical applications, and accessing library catalogs and stock. Thus, we hypothesized that students with better IT skills would perform better academically. Recent literature indicates that successful use of ICT positively affects and improves the performance of students (Díaz and Cano, 2019). Thus, in light of the above evidence, we considered IT skills as a component of this study and hypothesized that if students can utilize essential information technologies, their academic performance would improve. Past research investigated whether computer programming affects the reasoning skills, problem-solving abilities, and self-efficacy in mathematics of high school students. However, their findings did not confirm the hypothesis that computer programming substantially improves the problem-solving skills of students (Psycharis and Kallia, 2017). According to a Nigerian study, although many students are proficient users of various devices (desktop, laptop, and mobile devices), laptops are the favorite devices of students for academic work (Adegbilero-Iwari et al., 2021). Similarly, a study has shown a distinct difference between ICT self-efficacy and computer and information literacy (Hatlevik et al., 2018). Using the Bells University of Technology as a case study, an analysis was conducted to determine the ability of students to use educational technologies. Their results showed that the computing capacity of a large number of newly enrolled students (75 percent) is marginal, which would hurt their ability to deal with a computerized educational environment (Oshilalu and Emiri, 2018). Since students can access information anytime and from any place, ICT allows for greater consistency in education delivery (Johari et al., 2020).

#### Reading and Writing Skills

Fabulous readers are confident about their reading abilities and academic skills. They can read fast and understand what they are reading; they do not face punctuation, grammar, and spelling issues. They can appreciate the meaning of a text at first reading. Additionally, finding information effectively through reading is the quality of great readers and astute academics. Expert writers can quickly write their ideas and use the best words to develop quality material full of information without copying. Furthermore, people endowed with such skills are confident and comfortable while taking notes in lectures.

Similarly, successful writers can use dictionaries and thesauruses, they enjoy writing, and they find it convenient to describe what they say. Past literature, for example, indicated that cognitive reading skills have much effect on the performance of the student and are essential in performing the combined writing assignment than cognitive listening skills (Cheong et al., 2018). The advanced error factor seems more responsible than the essential error factor for reading, writing, and oral language skills (Choi et al., 2017). The core concept of the interactive dynamic literacy model is that reading and writing are interconnected and improve together, owing to a familiar constellation of skills and knowledge (Kim, 2020). As cognitive tasks in reading, writing, and mathematics, executive functions such as responsive inhibition, working memory, and mental flexibility are also necessary (Taghizadeh et al., 2017). There are positive associations between the reading habits and learning outcomes of students for academic writing, grammar skills and learning outcomes of students for academic writing, creative thinking of students, and learning outcomes of students for academic writing.

Similarly, there is a positive relationship between the reading habits, grammar skills, and creative thinking of students on the one hand and the student learning outcomes for academic writing on the other hand (Sukesi et al., 2019). Reading helps students improve their writing skills by inspiring them, expanding their vocabulary, and improving their grammatical structure (Mohammad Abdullah Attiyat, 2019). Thus, it can be said that some primary literature suggests that reading and writing are essential skills that play an invaluable role in various academic contexts. Still, in this research, we hypothesized that students would perform better academically if they have exceptional reading and writing abilities.

#### Resilience

Resilience is the ability to apply the experiences of a person during a wide range of complex circumstances and challenges. Persons with resilient skills can adapt their thinking to the issues at hand. Similarly, a person with resilient skills can deal with unpredictable situations and solve challenges as they occur. A recent study mentioned that resilience is the ability of the experienced person to overcome adversity and grow more robust in the face of challenges (Thomas and Asselin, 2018). In the relationship between depression and academic performance, stability acts as a mediator (Hart, 2019). Support from family and friends, academic self-efficacy, resilience, and engagement play significant roles in forecasting academic performance (Safaee et al., 2019). Good emotions predict both self-motivation and stability in physical education classes (Trigueros et al., 2019). An analysis revealed that while trait resilience scores predict the use of emotional regulation strategies, changes in stress and trait resilience do not predict variance in academic performance during the semester (Pendergast, 2017).

According to another examination, there is a substantial relationship between the levels of resilience of students and their academic performance. They also discovered that early adolescents are less resilient than late adolescents. Furthermore, they also proposed that effective counseling interventions should be created to improve the resilience capacities and academic skills of adolescents, resulting in improved academic performance and the ability to pursue the profession of choice and the capabilities of an individual (Rao and Krishnamurthy, 2018). A research discovered a significant positive association between academic resilience and self-efficacy with intellectual engagement, resilience, and self-efficacy. Furthermore, it suggested that academic self-efficacy is a mediating factor between academic resilience and academic engagement of students (Babajani Gorji et al., 2019). A methodological resilience model of nursing students discussed the value of promoting resilience in medical education (Thomas and Asselin, 2018). According to the preceding literature, resilience is an essential trait that can be useful in various situations. However, in this study, we hypothesized that students who can manage challenging conditions and utilize their resilience capacity during a period of academic stress would perform academically well in educational settings.

## Method

### Sampling

Few studies have examined the significance of IT skills, information-seeking habits, reading and writing ability, and resilience in academic contexts. For example, the research found that the information literacy abilities of graduate students needed to be improved (Korobili et al., 2011). Data from the postgraduate students of the University of Benin (Uniben) revealed a substantial difference in academic activity between users and non-users of ICT facilities. Their findings demonstrated that ICT has a favorable influence on student performance (Osagie et al., 2019). Similarly, data analysis of graduate students in four programs of an education department at a northeastern U.S. university revealed that students were positive about the use of computers in their graduate work and utilized computers often for various academic objectives (Shaw and Giacquinta, 2000). Graduates from a number of departments took part in the study. According to the findings of the research, their fundamental computer literacy levels were shown to be inadequate (Dincer, 2016). Another research findings showed that reading had a positive impact on the writing of students (Zainal et al., 2011). One element that may impact the move of new graduates to practice is resilience (Meyer and Shatto, 2018).

According to the preceding literature, previous researchers mostly concentrated on graduate students and investigated the impact of each component independently. While in this study, we offered a single platform for investigating the relevance of IT abilities, information-seeking behaviors, reading/writing ability, and resilience, in the contexts of perceptions of graduate students. We used a survey appraisal technique in this study to evaluate the association between different predictors and the academic performance of students. The included variables were culled from various observational studies, and the analytical data were measured using a Likert scale. Four hundred and fifty questionnaires were sent to postgraduate students, with 288 participants responding. The questionnaire of this study asked about five distinct factors and control variables: age, gender, marital status, and family income. The sample characteristics are shown in Table 1. The data of this study were obtained from students with diverse academic departments in three mega universities in the terrain of Islamabad, Pakistan.

**Table 1:** Characteristic of the demographic data.

**Variables**	***N***	**%**
**Age (years)**		
20–24	169	58.7
25 and above	119	41.3
**Gender**		
Male	188	65.3
Female	100	34.7
**Marital status**		
Single	237	82.3
Married	51	17.7
**Family income**		
15,000–25,000 PKR	102	35.4
26,000–35,000 PKR	137	47.6
36,000 PKR and above	49	17.0

### Measures

The two measurements, namely, hard IT skills and reading/writing abilities, were based on the individual learning profile scale (Pulford and Sohal, 2006). We adopted 12 original items of reading/writing and five original items of hard IT skills, while they were scored on a 4-point rating scale (i.e., 1—never, 2—sometimes, 3—mostly, and 4—always). The following two factors, information seeking and resilience, have been obtained from lifelong learning and cooperative education scales, respectively (Drewery et al., 2016). We used three items of information seeking and three resilience items in original form. At the same time, these two measures were assessed on a five-point Likert scale varying from (1—strongly disagree) to (5—strongly agree). The Personal Evaluation Inventory (PEI) scale was used to determine the dependent factor of academic performance (Shrauger and Schohn, 1995). We included seven original items of academic performance and measured with a five-point Likert scale (1—strongly disagree to 5—strongly agree).

### Data Analysis

The data were analyzed using several distinct steps to assess the impact of predictor variables on the academic performance of students. First, an internal consistency evaluation was conducted to assess reliability. Second, Pearson's correlations for all pairs of variables have been calculated. Third, hierarchical multiple regression analysis approaches were used to determine the effect of the conceptual construct on the dependent variable.

#### Psychometric Properties of Variables

To assess the internal consistency among the items of each variable, a reliability test was performed. The components included were those rated 0.7 or above. These results imply that the variables are accurate and adaptable. Because of these conclusions, we continued with the study. Table 2 displays the psychometric properties and descriptive statistics of embedded variables.

**Table 2:** Psychometric properties of the predictor and dependent variables.

**Variables**	**No. of items**	**Alpha**	**Means**	**SD**
Academic performance	7	0.91	3.96	0.75
Information seeking	3	0.82	3.86	0.81
Basic IT skills	5	0.80	3.93	0.78
Reading and writing skills	12	0.92	3.87	0.78
Resilience	3	0.74	4.03	0.74

#### Correlation Analysis

The study exemplified some considerations that are part of the investigation. In the current test, academic performance has been selected as the subordinate predictor. At the same time, age, gender, marital status, family income, information seeking, IT skills, reading/writing abilities, and resilience were the predicted variables. As a result, we only explained these sets of variables within the test with statistically meaningful relationships (see Table 3).

**Table 3:** Correlation among pairs of variables.

	**AP**	**IS**	**IT**	**R&D**	**RES**	**Age**	**GDR**	**MS**	**LFI**	**HFI**
Academic performance (AP)	1.00									
Information seeking (IS)	0.81**	1.00								
Basic IT skills (IT)	0.62**	0.49**	1.00							
Reading and writing skills (R&D)	0.69**	0.69**	0.43**	1.00						
Resilience (RES)	0.62**	0.66**	0.33**	0.55**	1.00					
Age	0.08	0.10	0.03	0.07	0.01	1.00				
Gender (GDR)	−0.05	−0.04	−0.11	0.03	0.03	0.03	1.00			
Marital status (MS)	−0.01	−0.04	−0.08	0.00	0.01	0.02	0.06	1.00		
Low family income (LFI)	−0.08	−0.09	0.04	−0.08	−0.10	−0.09	0.02	−0.06	1.00	
High family income (HFI)	0.04	0.05	−0.02	0.01	0.08	−0.06	0.04	0.09	−0.34**	1.00

N = 288, *p < 0.05, **p < 0.01.

The based factors, information seeking, IT skills, reading/writing skills, and resilience, reported positive relationship (0.81, *p* < 0.01; 0.62, *p* < 0.01; 0.69, *p* < 0.01; 0.62, *p* < 0.01) with academic performance. The first-best connections between information seeking and academic performance were established. Essentially, reading and writing abilities built up a second-best positive relationship with academic performance. Resilience is the third-best emphatically significant indicator that contains a positive relationship with academic performance. Basic IT skills build a less strong association with academic performance than information seeking, reading and writing, and resilience. During the study, basic IT skills, reading/writing, and resilience established a close relationship with information seeking (0.49, *p* < 0.01; 0.69, *p* < 0.01; 0.66, *p* < 0.01). These new relationships show that as the capacities of students for IT skills, reading/writing, and resilience increase, so would their information-seeking abilities. On the contrary, reading/writing and resilience built moderately positive relationships with IT skills (0.43, *p* < 0.01; 0.33, *p* < 0.01). This indicated that if reading/writing and resilience abilities of students increased in an academic environment, it would also increase their IT skills. During this study, resilience and reading/writing have formed a positive relationship (0.55, *p* < 0.01).

On the contrary, a negative association between low and high family income was discovered (−0.34, *p* < 0.01). All affiliations were extended from direct to solid, proposing that multicollinearity was improbable to be a concern. All the indicating factors were related to academic performance, recommending that the data were precisely associated with the subordinate variable for examination.

#### Forward Multiple Regression Analysis

Hierarchical regressions analysis was carried out to confirm the hypothetical model. The study, led by the theoretical model, was carried out to assess the interfaces between the components. The standardized and non-standardized regression coefficients have been shown, and the measurably relevant coefficients were examined.

A hierarchical multiple regression examination was carried out to discover the impacts of the independent variables on the academic performance of postgraduate students. The SPSS software was utilized to calculate the regression of the models, whereas the factors were entered into the investigation in four distinct steps. In the first stage of hierarchical regression, information seeking and age factors entered the analysis. The model was entirely accurate [*F*_(2,285)_ = 269.20, *p* < 0.0005], and information-seeking along with the control variable accounted for 65% of the difference in the academic performance of students. These elements significantly added to the individuality of the model (see Table 4).

**Table 4:** Hierarchical regression analysis, effects of predictors on academic performance.

	**ANOVA Sig**.	***R*^**2**^**	***R*^**2**^ change**	***F***	***F* change**	***B***	**SE**	**β**	***t***	**Sig**.
**Model 1**	0.000	0.65	0.65	269.20	269.20					
Information seeking						0.76	0.03	0.81**	23.10	0.000
Age						0.00	0.05	0.00	−0.06	0.954
**Mode 2**	0.000	0.72	0.07	181.37	33.03					
Information seeking						0.62	0.03	0.66**	18.21	0.000
IT skills						0.28	0.04	0.29**	8.09	0.000
Age						0.00	0.05	0.00	0.10	0.920
Gender						0.00	0.05	0.00	0.07	0.944
**Model 3**	0.000	0.74	0.02	133.86	11.62					
Information seeking						0.50	0.04	0.54**	12.05	0.000
IT skills						0.26	0.03	0.27**	7.65	0.000
Reading and writing						0.19	0.04	0.20**	4.66	0.000
Age						0.01	0.05	0.00	0.06	0.949
Gender						−0.02	0.05	−0.01	−0.41	0.685
Marital status						0.06	0.06	0.03	1.02	0.308
**Model 4**	0.000	0.75	0.01	93.41	3.99					
Information seeking						0.43	0.05	0.46**	9.36	0.000
IT skills						0.27	0.03	0.28**	7.84	0.000
Reading and writing						0.17	0.04	0.18**	4.10	0.000
Resilience						0.14	0.04	0.14**	3.34	0.001
Age						0.01	0.05	0.01	0.30	0.763
Gender						−0.03	0.05	−0.02	−0.62	0.537
Marital status						0.05	0.06	0.03	0.85	0.395
Low family income						−0.02	0.05	−0.01	−0.36	0.723
High family income						0.03	0.06	0.01	0.42	0.676

*p < 0.05, **p < 0.01.

Similarly, in the second stage, IT skills and gender were added to the analysis, while information seeking and age already existed. Thus, in the second model, information seeking, IT skills, age, and gender accounted for 72% of academic performance differences. At the same time, the significance of the second model was shown by the ANOVA table [*F*_(4,283)_ = 181.37, *p* < 0.0005]. Reading/writing skills and marital status were added to the model in the third phase, and the overall model was found to be significant with information-seeking behavior, IT skills, reading/writing, age, gender, and marital status [*F*_(6,281)_ = 133.86, *p* < 0.0005]. As a result, the integrated predictors, including information-seeking behavior, IT skills, reading/writing ability, age, gender, and marital status, account for 74% of the variance in academic performance. In the fourth step of the study, the model was found significant [*F*_(9,278)_ = 93.41, *p* < 0.0005]. This stage incorporated resilience and family income into the model to determine the difference in academic performance. As data on family income were collected in three batches, dummy variables were created for data distributions in three layers: low family income, average family income, and high family income. As a result, the integrated predictors, which included information seeking skills, IT skills, reading/writing skills, resilience, age, gender, marital status, and family income, explained 75% of the variation in academic performance.

Model one had an “*R*^2^ change” of 65%, while model two had a change of 7%. Similarly, the “*R*^2^ change” for model three was 2%, and for model four, it was 1%. These results indicated that information seeking and age were associated with a 65% improvement in academic performance. Based on the results, we might infer that the most significant factors in enhancing the academic performance of students are information seeking and age. The 75% *R*^2^ for model four allows us to assume that it is the best model of all other models when all variables are considered.

The fourth uniform coefficient (beta value) of the model revealed that academic performance was affected by the information-seeking abilities (β = 0.46, *p* < 0.01). The findings indicated that the academic performance of students would improve if they engaged in an information-seeking activity. This also concludes that students who lack information-seeking skills during their school years could never perform excellent academically. Similarly, basic IT skills have influenced academic performance, with an optimistic and essential standardized coefficient (β = 0.28, *p* < 0.01). The findings revealed the significance of IT capabilities in transforming perceptions toward academic performance in educational settings. As a result, we could agree that students with IT competence would perform well-academically, while students lacking IT skills will not perform well-academically.

Similarly, reading/writing abilities have influenced the academic performance of students in educational settings (β = 0.18, *p* < 0.01). This result showed that students with reading and writing abilities would perform excellently compared with students who do not have this ability. Similarly, resilience significantly affected the academic performance of students in educational settings (β = 0.14, *p* < 0.01). Based on these findings, we might infer that students who can cope with intellectual tension during their campus tenure would perform well-academically. In contrast, students who lack resilience in the academic atmosphere would perform poorly.

## Discussion

In this research, the self-efficacy theory of Bandura (1997) was used to choose the information-seeking behavior, where confidence in the abilities of an individual is an essential concept. We had predicted that if students believe in finding the correct information for their academic needs, they will perform remarkably well-academically. To put the assumptions to the test, we used correlation and regression analysis on existing data. As a result, information-seeking behaviors and age emerged as the critical collection of variables in this study. These two variables have a significant impact on the academic performance of Pakistani university students. On the contrary, the research looked at the information-seeking behaviors of programming learners in online discussion forums and how to provide visual navigational support to assist them in finding information. According to the findings, paying attention to history can contribute to additional reading events, leading to potential learning activities (Lu and Hsiao, 2017).

Furthermore, a study at the University of Dhaka identifies patterns of information-seeking activity among law students in digital environments. Their findings revealed that students prefer electronic information over printed information (Das and Jadab, 2017). According to recent research findings, the five personality traits were strongly linked to all dimensions of the information-seeking behavior of university students (Halder et al., 2020). Previous experiments have measured information seeking in diverse contexts and across multiple continents. However, this study found a strong positive association between the cumulative academic performance and information-seeking activities of students. Information seeking and age have had a significant and constructive impact on academic performance. Thus, the assumption of self-efficacy had proven authentic because the findings showed that people who can seek the correct information perform exceptionally well-academically.

We had predicted that if students believed in using basic technology for academic purposes, they would perform exceptionally well-academically. According to this theory, the self-efficacy theory of Bandura (1997) was used to endorse the fundamental IT skills. According to this theory, belief in the ability of an individual is a crucial idea. We added IT skills and gender to the second model, which already had information-seeking skills and age. As a result, the two causes, namely, IT skills and information-seeking behaviors, and the two control variables, namely, age and gender, significantly impact the academic performance among Pakistani university students. On the contrary, a study showed that attitude toward IT has a significant negative association with academic performance (Johari et al., 2020).

In comparison to this, we discovered a significant relationship between IT skills and academic performance. Furthermore, we found that information-seeking behaviors, IT skills, age, and gender all had a positive impact on academic performance. Similarly, another study advised that students seeking entry to the Bells University of Technology receive hands-on computer training sessions (Oshilalu and Emiri, 2018). At the same time, research showed that ICT literacy standards significantly correlate with academic performance in Arts and Social Sciences, Health Science, Management Science, and Science and Technology e-learners, but not in Agriculture, Education, or Law e-learners (Aboderin, 2019). Students who were instructed with ICT had a better academic performance on Christian Religious Studies (Ikwuka and Adigwe, 2017). Previous studies have tested information seeking in various contexts; however, in this research, self-efficacy theory about the abilities of students to use essential technology during academic need was proven. The results revealed that people who can use basic computers perform exceptionally well-academically.

The self-efficacy theory of Bandura (1997) was used to endorse the students reading and writing skills in this study. According to this theory, belief in the ability of an individual is a crucial idea. We had predicted that if students believe in reading and writing, they will perform exceptionally well-academically. We added reading/writing skills and marital status to the third model (see Table 4, model 3), which already had information seeking, IT skills, age, and gender. As a result, the three factors, namely, information seeking, IT skills, and reading/writing skills, and the three control variables, namely, age, gender, and marital status, were significant in the academic performance among Pakistani university students. While according to a qualitative study, the majority of participants had a pessimistic view of in-school reading. Similar findings were taken from the effects of in-school writing because most participants felt constrained and under pressure during the writing process. Reading in school elicited negative feelings such as feeling obligated, lonely, and denied and seeing the activity as pointless. Out-of-school writing activities were determined to foster intrinsic inspiration, improved self-expression of anxiety and happiness of an individual, and developed digital writing skills (Bal, 2018).

Thus, reading interventions can help students improve their writing skills (Graham et al., 2018). Participants in the qualitative analysis reported that they used the skills they learned in school to assist them with reading and writing outside of the classroom. Participants indicated that their academic literacy abilities improved their non-academic skills, but some were uneasy with online non-academic reading and writing due to linguistic or cultural differences (Wang, 2020). Previous research has evaluated reading and writing in several contexts; however, in this study, the theoretical hypothesis of self-efficacy regarding the reading and writing abilities of students during the academic year has been confirmed, and the findings showed that individuals with excellent reading and writing abilities performed incredibly well-academically.

Life skills education is described as the “psychosocial skills required to deal with the demands and challenges of everyday life” (World Health Organization, 1999). We followed the life skills framework listed by the WHO, in which they emphasized one fundamental aspect, the ability to deal with stress, which is known as resilience abilities. In this way, we had predicted that students who can deal with academic tension would perform better academically. Resilience and family income were applied to the fourth model (see Table 4, model 4), which already included information seeking, IT expertise, reading/writing skills, age, gender, and marital status. As a result, the four factors, namely, information seeking, information technology skills, reading/writing skills, and resilience, as well as the four control variables, namely, age, gender, marital status, and family income, were discovered to have a significant effect on the academic performance among Pakistani university students. Past literature indicated that positive thinking improves the resilience and academic performance of students (Omidi and Rasoli, 2019).

Similarly, academic meaning has a significant relationship with academic performance, and academic resilience has a substantial connection with theoretical significance. Academic strength has a meaningful relationship with the academic performance of female high school students in Jafari et al. (2020). While teacher leadership predicted academic resilience and motivation, intellectual strength predicted burnout but not literary success; academic motivation predicted burnout but not academic performance; and burnout predicted academic stability but not academic performance (Trigueros et al., 2020). In this study, we have found a strong association between resilience and academic performance.

Additionally, we found that all the variables, including resilience in the regression analysis of the fourth model, influenced the academic performance of students (see Table 4, model 4). Previous research tested the strength in various contexts; however, the current analysis validated the theoretical hypothesis of life skills about the ability of students to use resilience skills during academic stress situations. The results revealed that individuals with outstanding skills to use resilience consistently perform well-academically.

## Conclusions

The core set of variables of this study emerged as information-seeking behaviors and age. These two factors have a significant influence on the academic performance of Pakistani university students. This research discovered a meaningful, beneficial relationship between the cumulative academic performance and information-seeking behaviors of students. Both information seeking and age have had a significant and valuable impact on academic performance. The study revealed that if students could find accurate information about their educational needs, they would perform well-academically compared with students who cannot find precise information about their academic needs.

The two factors, IT skills and information-seeking habits, and the two control variables, age and gender, have significantly affected the academic success of Pakistani university students. We discovered a strong relationship between IT skills and academic performance. The findings showed that people who can use simple computers perform exceptionally well at the university. Thus, it has been discovered that if students can use a computer for academic purposes, i.e., the ability to use spreadsheets, run and manage databases, make presentations, practice statistical tools, and access online library catalogs and inventory, they will perform better academically.

We discovered a significant and constructive relationship between reading/writing and academic performance. We introduced reading/writing abilities and marital status to the third model (see Table 4, model 3) and discovered that they significantly impact academic performance. These findings revealed that students who can read quickly and correctly, have consistency in grammar and punctuation, locate the correct information, use a dictionary and thesaurus, and enjoy writing would perform admirably academically well. Similarly, students who can write their ideas by finding suitable vocabulary without copying perform better academically.

Academic success and resilience have a deep and constructive relationship (see Table 3). In addition, in the fourth model (see Table 4, model 4), we added resilience and family income. It was discovered that resilience and family income demonstrated a significant gap in academic performance. These results revealed that students with the ability to use a wide range of knowledge might be stacked at a particular stage, can use self-thinking or ideas when confronted with learning challenges, and can find the correct information would perform excellent academically. Similarly, students who can use self-thinking and personal experiences when tackling unexpected problems could perform outstanding academically.

### Limitations

The PEI assesses particular self-esteem and self-confidence in many elements of life of an individual, i.e., speaking in public, academic performance, physical appearance, social interactions, athleticism, and general confidence and mood state (Shrauger and Schohn, 1995). Hence, we chose a domain that focuses on the confidence of students in their academic performance in educational contexts. We did not evaluate student grades and exam results. We have only focused on academic self-confidence which can both improve and reduce performance. Similarly, the study found that increases or decreases in self-confidence were rated as either increasing or decreasing performance (Hanton and Connaughton, 2002). We gathered data from government universities but did not include private sector institutions in the current report. Data from postgraduate candidates were obtained for this study, but no data from undergraduates were used. Similarly, in this study, we gathered data from Pakistani students but did not include data from other ethnic groups. If we consider private sector institutions, undergraduate students, and other ethnic groups, the findings can vary.

### Recommendations

The educationists need to ensure the awareness of how students should obtain relevant information about academic requirements and the most recent resources. Educators should direct students to the web pages and tools of the library or recommend any books related to the work of the class. Educators need to hold extra elective credits to apply the primary technological curriculum for all newly registered students. Faculty professionals need to direct students to learn the spreadsheet, MS Word, lab numerical tools, PowerPoint, and digital inventory and library access processes. To stimulate the resilience skills of students, policymakers should train university educators on elevating emotions, commitment for ambitions, motivating multifaceted situations, and using these motivational strategies among students.

### Future Research

Future researchers should focus on faculty training and skills to ensure that they can increase the overall academic performance of students. Researchers should investigate how the teaching background of mentors affects the academic success of students. Future researchers should investigate how university rankings and educational facilities affect the academic performance of students.

## Data Availability Statement

The raw data supporting the conclusions of this article will be made available by the authors, without undue reservation. Requests to access the datasets should be directed to muhammadmiraj85@yahoo.com.

## Ethics Statement

The studies involving human participants were reviewed and approved by Biomedical Ethics Committee of Xi'an Jiaotong University, China. Written informed consent for participation was not required for this study in accordance with the national legislation and the institutional requirements.

## Author Contributions

MM and LC designed and established the frame for this research. MM was responsible for the data conduction and compilation. RMS and RO-B made the study logical, recommended appropriate literature searches, and helped with draft proofreading, analysis, and interpretation of results. RR contributed to the manuscript editing and compilation and also took part in the interpretation of results and theoretical framework compilation. All authors contributed to the article and approved the submitted version.

## Conflict of Interest

The authors declare that the research was conducted in the absence of any commercial or financial relationships that could be construed as a potential conflict of interest.

## Publisher's Note

All claims expressed in this article are solely those of the authors and do not necessarily represent those of their affiliated organizations, or those of the publisher, the editors and the reviewers. Any product that may be evaluated in this article, or claim that may be made by its manufacturer, is not guaranteed or endorsed by the publisher.
