# Supplementary material for: How Information-Seeking Behavior, Essential Technologies, and Resilience Enhance the Academic Performance of Students
Source: Front Psychol. 2021 Aug 26;12:651550. doi: 10.3389/fpsyg.2021.651550 (PMC8428236; doi:10.3389/fpsyg.2021.651550)
Supplement: Supplementary file 1 [file Table_1.docx]

**APPENDIX- A**

| **Table 1: Details of Items and scales used** |
| --- |
| **Self Confidence in academic performance: This subscale is based on the Personal Evaluation Inventory (PEI) scale. The PEI was designed to assess confidence, or sense of competence/skill, across a range of domains important to students (**[**Shrauger and Schohn, 1995**](#_ENREF_78)**). We used the 1 to 5 scale i.e. “one-strongly disagree to five-strongly agree”. The Negative signs (-) indicated negative worded questions.** |
| 1. Academic performance is an area in which I can show my competence and be recognized for my achievement. 2. I frequently wonder whether I have the intellectual ability to successfully achieve my vocational and academic goals. (-) 3. I have recognized that I am not as good a student as most of the people with whom I am competing. (-) 4. It’s bother me that I don’t measure up to others intellectually. (-) 5. When I take a new course, I am usually sure that I will end up in the top 25% of the class. 6. When I have to come through on important tests or other academic assignments, I know that I can do it. 7. I seek out activities that are intellectually challenging, because I know I can do them better than most people. |
| **Information seeking and resilience: The following items relate to certain characteristics (e.g. attitudes and behaviors) related to the student's learning experiences and are adapted from lifelong learning (**[**Drewery et al., 2016**](#_ENREF_27)**). We used the 1 to 5 scale i.e. “one-strongly disagree to five-strongly agree”.** |
| **Information seeking behavior:**   1. I am very good at seeking and retrieving information. 2. If I discover a need for information that I don’t have, I know where to go to get it. 3. I often know where to look for solutions to complex problems. |
| **Resilience:**   1. I can apply my knowledge across a variety of situations and problems. 2. I adapt my thinking to the problems at hand. 3. I can deal with the unexpected and solve problems as they arise. |
| **Individual Learning Profile (ILP) is a scale that measures students' confidence in their academic skills. It contains six aspects (Reading and Writing, Hard IT, Numeracy, Time Management, Speaking, and Easy IT) and is scored on a 4-point scale (i.e., 1-never, 2-sometimes, 3-mostly, and 4-always) (**[**Pulford and Sohal, 2006**](#_ENREF_64)**). We adopted Reading and writing and Hard IT domains of this scale.** |
| **Reading and writing skills:**   1. Are you confident about your reading skills? 2. Are you able to read fast and understand what you are reading? 3. Are you confident in the use of punctuation and grammar? 4. Are you able to make sense of a text on first reading? 5. Are you confident about your spelling? 6. Can you find information easily by reading? 7. Can you get your own ideas onto paper easily, and find the right words? 8. Can you put information into your own words without copying big chunks? 9. Are you confident about taking notes in lectures? 10. Are you confident about using a dictionary and/or thesaurus? 11. Do you enjoy writing? 12. Do you find it easy to explain what you mean (e.g. find the right words)? |
| **Hard IT Skill:**   1. Are you confident using computers for: Spreadsheets 2. Are you confident using computers for: Accessing library catalogues and stock 3. Are you confident using computers for: Databases 4. Are you confident using computers for: Presentations (e.g. PowerPoint) 5. Are you confident using computers for: Statistics |
